# Enhancer buffering protects dosage-sensitive housekeeping genes during vulnerable developmental transitions

**DOI:** 10.64898/2026.07.29.741571

**Published:** 2026-08-31

**Authors:** Chunyang Ni, Lucia Ichino, Tomek Swigut, Joanna Wysocka

## Abstract

Housekeeping genes maintain robust expression across cell types despite dynamic transcription factor fluctuations, yet their haploinsufficiency is associated with many tissue-specific developmental disorders. To understand this paradox, we focus on TCOF1, a broadly expressed regulator of rRNA synthesis, whose haploinsufficiency causes Treacher Collins syndrome (TCS). We show that transitional cranial neural crest cells (tCNCC) undergoing mesenchymal specification exhibit extreme dosage-sensitivity to TCOF1, but not to RNA polymerase I, explaining both cellular origins of TCS and prevalence of TCOF1 mutations in the disease. To maintain robust expression, TCOF1 deploys CNCC-specific enhancers that buffer against fluctuations in promoter-regulating factors, converting sensitive expression responses into threshold-protected outputs. Systematic promoter-enhancer coupling experiments demonstrate this principle generalizes across many dosage-sensitive housekeeping genes, whose promoters operate near saturation and only reveal their enhancer-dependency under suboptimal conditions. Thus, enhancers play a unique role at housekeeping promoters: rather than amplifying expression, they ensure its robustness during vulnerable developmental transitions.

## Full Text Availability

The license terms selected by the author(s) for this preprint version do not permit archiving in PMC. The full text is available from the preprint server.

